# “VID-KIDS” Video-Feedback Interaction Guidance for Depressed Mothers and Their Infants: Results of a Randomized Controlled Trial

**DOI:** 10.3390/bs15030279

**Published:** 2025-02-27

**Authors:** Panagiota D. Tryphonopoulos, Deborah McNeil, Monica Oxford, Cindy-Lee Dennis, Jason Novick, Andrea J. Deane, Kelly Wu, Stefan Kurbatfinski, Keira Griggs, Nicole Letourneau

**Affiliations:** 1 Arthur Labatt Family School of Nursing, Western University, 1151 Richmond St., London, ON NBA 5C1, Canada; 2 Alberta Health Services, Alberta Children’s Hospital Research Institute, Faculty of Nursing, Department of Community Health Sciences, Cumming School of Medicine, University of Calgary, 2500 University Drive NW, Calgary, AB T2N 1N4, Canada; debbie.mcneil@albertahealthservices.ca; 3 Department of Child, Family, and Population Health Nursing, University of Washington, 1410 NE Campus Parkway, Seattle, WA 98195, USA; mloxford@uw.edu; 4 Lawrence S. Bloomberg Faculty of Nursing, University of Toronto, 155 College Street, Toronto, ON M5T 1P8, Canada; cindylee.dennis@utoronto.ca; 5 Owerko Centre for Children’s Neurodevelopment and Mental Health, Alberta Children’s Hospital Research Institute, University of Calgary, 2500 University Drive NW, Calgary, AB T2N 1N4, Canada; jbnovick@ucalgary.ca (J.N.); andrea.deane@albertahealthservices.ca (A.J.D.); kgywu@ucalgary.ca (K.W.); stefan.kurbatfinski@ucalgary.ca (S.K.); keira.griggs@ucalgary.ca (K.G.); 6 Owerko Centre for Children’s Neurodevelopment and Mental Health, Alberta Children’s Hospital Research Institute, Faculty of Nursing, Cumming School of Medicine, Departments of Pediatrics, Psychiatry and Community Health Sciences, University of Calgary, 2500 University Drive NW, Calgary, AB T2N 1N4, Canada; nicole.letourneau@ucalgary.ca

**Keywords:** video-feedback, positive parenting intervention, postpartum depression, infant development, randomized controlled trial

## Abstract

VID-KIDS (Video-Feedback Interaction Guidance for Depressed Mothers and their Infants) is a positive parenting programme comprising three brief nurse-guided video-feedback sessions (offered in-person or virtually via Zoom) that promote “serve and return” interactions by helping depressed mothers to be more sensitive and responsive to infant cues. We examined whether mothers who received the VID-KIDS programme demonstrated improved maternal–infant interaction quality. The secondary hypotheses examined VID-KIDS’ effects on maternal depression, anxiety, perceived parenting stress, infant developmental outcomes, and infant cortisol patterns. A parallel group randomized controlled trial (*n* = 140) compared the VID-KIDS programme to standard care controls (e.g., a resource and referral programme). The trial was registered in the US Clinical Trials Registry (number NCT03052374). Outcomes were assessed at baseline, nine weeks post-randomization (immediate post-test), and two months post-intervention. Maternal–infant interaction quality significantly improved for the intervention group with moderate to large effects. These improvements persisted during the post-test two months after the final video-feedback session. No significant group differences were detected for secondary outcomes. This study demonstrated that nurse-guided video-feedback can improve maternal–infant interaction in the context of PPD. These findings are promising, as sensitive and responsive parenting is crucial for promoting children’s healthy development.

## 1. Introduction

Perinatal mood disorders, such as postpartum depression (PPD), are the most common complication following childbirth, with PPD prevalence rates ranging from 15% to 20% in developed countries (Dennis et al., 2017; Fawcett et al., 2019; Liu et al., 2022). Maternal depression can inadvertently contribute to children’s heightened risk for future mental health and behavioural problems since depressive symptoms can undermine the quality of early relationships (Center on the Developing Child at Harvard University, 2009). Since maternal sensitivity and responsiveness (i.e., a mother’s ability to notice, interpret, and respond to infant communication) are the primary behavioural mechanisms influencing children’s development (National Scientific Council on the Developing Child, 2012; Mateus et al., 2021), these are also key modifiable intervention targets for women with PPD (Letourneau et al., 2017; Tryphonopoulos & Letourneau, 2020).

### 1.1. Impact of PPD on Parent–Infant Relationships and Child Development

PPD has profound consequences for both mothers and their infants, negatively impacting maternal–infant interaction quality and child development (Garner et al., 2012; Logsdon et al., 2006; Shonkoff et al., 2012; Slomian et al., 2019). Aptly described as “the thief that steals motherhood” (Beck, 1996, p. 736), PPD diminishes mothers’ sensitivity and positive responsiveness toward infant cues (Field et al., 2010; J. H. Goodman et al., 2015). Infants perceive this impaired interaction as stressful, triggering cortisol release, which, at persistently elevated levels, inhibits neurodevelopment during critical periods (Ashman et al., 2002; Letourneau et al., 2011b). PPD’s impact on children is multifaceted, affecting social, emotional, motor, intellectual, cognitive, and immunological development (Field, 2010; S. H. Goodman & Gotlib, 1999; Luoma et al., 2001; Thompson & Fox, 2011). Infants of mothers with PPD exhibit less sociability, increased fearfulness of strangers, more frustration, separation anxiety, and insecure attachments (Forman et al., 2007; Garner et al., 2012; Poobalan et al., 2007; Shonkoff et al., 2012). These effects can persist into adolescence and early adulthood, with increased rates of major depressive disorder, anxiety disorders, and alcohol dependence (Garner et al., 2012; Shonkoff et al., 2012). The negative impact of PPD on child development is particularly marked when depression persists beyond the first few months postpartum, highlighting the importance of early intervention and treatment (Slomian et al., 2019).

### 1.2. Supporting Parenting in the Context of PPD

Psychological interventions, particularly short-term cognitive, behavioural, and interpersonal psychotherapies, have demonstrated significant efficacy in reducing maternal depressive symptoms (O’Connor et al., 2019). However, accessibility to these treatments can be hindered by systemic barriers (e.g., unclear referral pathways and shortage of specialized healthcare providers, especially in rural areas) and patient-specific factors such as lack of time, difficulty finding childcare during appointments, and the pervasive stigmatization of PPD (Dennis et al., 2024). Treatment approaches that solely target maternal depressive symptoms do not necessarily translate to concomitant improvements in the quality of mother–infant interactions or child developmental outcomes (Forman et al., 2007; Poobalan et al., 2007; Richards et al., 2024; Tsivos et al., 2015). A more comprehensive strategy addressing both maternal mental health and parenting may be necessary to promote optimal outcomes for both mother and child (Horowitz et al., 2013).

Despite the presence of depressive symptoms, mothers who maintain positive interactions and emotional connections with their infants may help protect their children from some of the negative impacts of PPD (Horowitz et al., 2013). Positive parenting programmes that increase maternal–infant interaction skills by promoting sensitive and responsive exchanges may help to improve parenting self-efficacy and competence in the context of PPD (Adina et al., 2022; Horowitz et al., 2013; Jung et al., 2007). A recent meta-analysis of parenting interventions for perinatal mood disorders (Adina et al., 2022) reported a significant reduction in depressive symptoms in mothers, with a standardized mean difference (SMD) of −0.34, indicating a positive impact on maternal mental health. However, the same review reported that within the studies that met inclusion criteria, data on infant developmental outcomes were scarce, suggesting a need for further research to fully understand the impact of parenting interventions on infants.

### 1.3. Video-Feedback as a Modality for PPD Interventions

Meta-analyses have demonstrated that the most successful methods for improving parental sensitivity at the behavioural level are by incorporating direct feedback to parents through recording and playing back videos of parent–infant interaction (i.e., video-feedback) (Bakermans-Kranenburg et al., 2003; O’Hara et al., 2019). Video-feedback-based interventions support caregivers’ understanding of their infants’ nonverbal language (e.g., bids for caring attention and disengagement cues), which, in turn, promotes sensitive and responsive interactions (Beebe, 2003; O’Hara et al., 2016). Video-feedback has the advantage of being visually concrete yet “distant” because the action and the feedback are not concurrent; this temporal separation helps mothers maintain an element of objectivity that minimizes guilty feelings associated with any perceived lack of parenting skills (Beebe, 2003, 2005; O’Hara et al., 2016, 2019). Furthermore, video-feedback focuses on building parental competence rather than highlighting deficiencies, encouraging a growth mindset in developing parenting skills (Beebe, 2003, 2005). Thus, video-feedback interventions may be promising strategies for promoting positive parenting to ameliorate the harmful impact of PPD on the developing child.

Only two previous randomized controlled trials (RCTs) have evaluated Registered Nurse (RN) delivered video-based interventions designed to improve maternal–infant interactions in the context of PPD (Horowitz et al., 2001, 2013). Horowitz and colleagues (Horowitz et al., 2001) tested an interactive coaching programme (ICAP) designed to promote healthy responsiveness between mothers with depressive symptoms and their infants (*n* = 117). The ICAP consisted of six elements focusing on teaching mothers to identify and respond to infant cues, positioning, interaction techniques, modelling, reinforcement, and praise. A nurse delivered the intervention through three home visits at 3–5-week intervals when infants were aged 4–18 weeks. Significantly higher maternal–infant responsiveness, as measured by the Dyadic Mutuality Code (Censullo et al., 1987), was observed in the intervention group (*n* = 60). While the intervention did not aim to decrease depressive symptoms, no significant change in maternal mood was observed.

Horowitz and colleagues (Horowitz et al., 2013) also tested whether a relationship-focused behavioural coaching intervention (Communicating and Relating Effectively; CARE) increased maternal–infant relational effectiveness between depressed mothers (*n* = 134) and their infants. A nurse delivered the intervention over five home visits when infants were aged approximately 6 weeks to 6 months. CARE focuses on interpreting infant cues, mitigating problematic maternal behaviours, and promoting responsive maternal behaviours through social reinforcement. The efficacy of CARE was only partially supported, as both intervention and control groups showed improved mother–infant interaction quality and decreased depression severity, with no significant group differences. The authors posited that nurse attention (e.g., empathetic listening, social support) given to the control group likely confounded results, creating unintentional treatment effects.

### 1.4. Current Investigation

This study builds on a feasibility pilot (Tryphonopoulos & Letourneau, 2020), which tested VID-KIDS (Video-Feedback Interaction Guidance for Improving Interactions Between Depressed Mothers and their Infants). The VID-KIDS parent training programme consists of three nurse-guided video-feedback sessions targeting maternal sensitivity and responsiveness to promote “serve and return” interactions (i.e., reciprocal exchanges where the child initiates engagement and the caregiver responds appropriately). The pilot (*n* = 12) yielded promising results, demonstrating significant differences with large effects favouring the intervention group in overall maternal–infant interaction quality (*d* = 1.43) and reduced infant diurnal cortisol patterns (*d* = 0.5). Although maternal mood improved for both groups throughout the study, the intervention did not appear to impact depression significantly.

Findings from both studies by Horowitz et al. (2001, 2013), as well as the VID-KIDS pilot (Tryphonopoulos & Letourneau, 2020), suggest that nurse-led interaction guidance interventions may improve relationships between depressed mothers and their infants. However, further research is needed to establish the efficacy of video-feedback parenting interventions for PPD and what benefits may be conferred to mothers and their infants. VID-KIDS offers unique contributions to this field through its structured approach to video feedback using an intervention fidelity assessment tool, a longer 2-month post-intervention follow-up period, and an expanded scope of outcome measures, including parenting stress and infant development.

Accordingly, the primary aim of this study was to test the efficacy of the VID-KIDS programme in improving maternal–infant interaction quality (primary outcome). Secondary aims included exploring the impact of VID-KIDS on maternal depression, maternal anxiety, parenting stress, infant development, and infant cortisol patterns (secondary outcomes).

## 2. Materials and Methods

### 2.1. Trial Design

Between June 2017 and July 2022, we conducted a parallel group RCT comparing the VID-KIDS programme to standard care controls (e.g., a resource and referral programme). After baseline assessment, over the following 9 weeks, mothers randomized to intervention conditions received three RN-guided video-feedback sessions conducted at 3-week intervals. Control participants received three home visits (conducted in-person or virtually via Zoom) on the same schedule. Mothers in both the intervention and control groups received guidance and advice on topics of interest (i.e., infant oral care, addressing infant sleep issues, stress management, self-care), with mothers in the control group receiving equivalent time and interaction with the nurse. Outcomes for both groups were assessed at baseline, nine weeks post-randomization (immediate post-test), and two months post-intervention (delayed post-test). The study protocol was developed using the CONSORT Statement for RCTs (Schulz et al., 2010) and approved by the Conjoint Health Research Ethics Board at the University of Calgary (REB16-1811). The trial was registered in the US Clinical Trials Registry (NCT03052374). While VID-KIDS was initially designed as a home visiting programme, some participants received the intervention via the videoconferencing platform Zoom due to public health restrictions implemented during the COVID-19 pandemic.

### 2.2. Study Setting, Participant Recruitment and Enrollment

The VID-KIDS programme trial took place in Calgary, a socioeconomically and ethnically diverse metropolitan city with a population of approximately 1.4 million located in Alberta in western Canada (Statistics Canada, 2024). Participants were recruited in partnership with Alberta Health Services (AHS), the provincewide, integrated health system responsible for delivering hospital and community-based services. In Alberta, mothers attending Public Health Well Child Clinics (WCC) are routinely screened for PPD using the Edinburgh Postnatal Depression Scale (EPDS) (Cox et al., 1987) during infants’ vaccination appointments beginning at 2 months of age, and then again at 4 and 6 months. We funded a part-time AHS Public Health clerk to review screening records collected from the WCCs and contact potential participants to determine if they were willing to be contacted by VID-KIDS study staff for further information about the study. VID-KIDS study staff conducted the initial telephone screening, explaining the nature of the study, randomization procedures, and strategies to maintain participant privacy and confidentiality throughout the study. Eligible mothers were asked to read the study Letter of Information and, if interested, provide written consent via an online consent form. Once written consent was obtained, mothers were contacted by a VID-KIDS nurse interventionist to answer any questions about the study, reaffirm consent, and arrange an initial home visit.

### 2.3. Inclusion and Exclusion Criteria

Eligibility criteria were based on the following: (1) an EPDS cut-off of greater than 12 (suggesting probable PPD); (2) infant aged 2–6 months at baseline; (3) maternal age between 18 and 49 years; (4) availability for home visits for the duration of the programme (i.e., 16 weeks), and (5) willingness to be video-recorded. Participants were not excluded from the trial based on taking anti-depressants or using other interventions for PPD (i.e., counselling, psychotherapy). However, mothers receiving other parenting support services (e.g., Circle of Security programme, infant–parent psychotherapy) were excluded due to the potential for the dilution of intervention effects. Prospective participants who declined to participate or were screened but found ineligible were offered literature on infant cues and behaviours and as required, were referred to Public Health or other local family resources.

### 2.4. Randomization and Blinding

Before recruiting participants, a randomization scheme was created using a random number-generating website (https://www.randomizer.org/) by a research programme staff member who was not associated with the VID-KIDS trial. The staff member placed an opaque envelope containing the randomization results into individual participant file folders stored in a locked file cabinet. Once baseline measures were collected, the RN interventionist opened the opaque envelope, revealing group allocation. Due to the face-to-face nature of the intervention, neither the RN interventionist nor the participants were blind to group allocation. However, all measures were either self-report or scored by an assessor who was naive to group allocation.

### 2.5. Power Calculation and Sample Size

Statistical power analysis used effect sizes for maternal–infant interaction quality from the VID-KIDS pilot (Tryphonopoulos & Letourneau, 2020) and other published studies (Drummond et al., 2008; Letourneau et al., 2011a), ranging from *d* = 0.5 to 1.43. G*Power3 (Faul et al., 2007) analysis revealed that 51 mother–infant pairs per group would be required to obtain 80% power, given a conservative medium (*d* = 0.5) effect size and alpha = 0.05.

### 2.6. Description of the VID-KIDS Intervention

The development of the VID-KIDS programme was informed by Kathryn Barnard’s Child Interaction Theory, also known as the Barnard Model (Barnard & Eyres, 1979). This theory characterizes optimal maternal–infant interaction as mutually adaptive and dependent upon caregivers and infants fulfilling certain responsibilities. Parents must demonstrate affectionate caregiving by being sensitive and responsive to infant needs, and infants must provide clear cues so that caregivers can respond appropriately. Contingency (i.e., responding to an infant’s smile with a smile) is central to Barnard’s Model since infants develop a sense of self-efficacy when caregivers respond reciprocally to their behaviour. Building on the principles of mutual adaptation and reciprocal interaction, we created VID-KIDS to offer mothers affected by PPD a strengths-based approach to improving sensitivity and positive responsiveness toward their infants (Tryphonopoulos & Letourneau, 2020). Video-feedback sessions lasted approximately 45–60 min and followed an eight-step protocol:

1. Icebreaking. The nurse interventionist initiates rapport (e.g., asks questions about the baby and inquires how the mother is coping).
2. Engagement and Disengagement Cues. Using BabyCues: A Child’s First Language Cards^®^ (Seattle, WA, USA) nurses review 59 photos illustrating common infant behavioural cues (e.g., engaging and disengaging).
3. Selecting Teaching Activity. Using the Parent–Child Interaction Teaching Scale (PCITS) (Sumner & Spietz, 1994) protocol, mothers select and perform a structured teaching task with their infants.
4. Recording Interaction. Dyads are observed and recorded for approximately 5 min.
5. Initial Viewing. Specific feedback is not provided; instead, mothers are asked to reflect on and point out any infant cues they recognize. The nurse documents their observation of infant cues (engagement and disengagement) for later discussion.
6. Second Viewing. The nurse and mother co-view the recording, with opportunities provided to review selected portions emphasizing sensitivity and responsiveness. The nurse provides feedback, including praise to reinforce maternal sensitivity, information on infant cues, appraisal of maternal responses to infant distress, and use of cognitive growth-fostering language (e.g., teaching the infant about colours by describing a toy).
7. Third Viewing. This step integrates all the concepts discussed with mothers in the previous screenings, using positive reinforcement to emphasize optimal aspects of sensitivity and responsiveness and constructive feedback to suggest areas for growth. A balance between positive and constructive comments is maintained to encourage parenting self-efficacy and minimize guilt.
8. Post-viewing Debrief. The session concludes with a general discussion of whatever interests the participant. Mothers are also encouraged to reflect on their observations of their infants’ engagement and disengagement cues in between video-feedback sessions.

Prior to concluding the session, the nurse offers the mother a summary of four strengths (e.g., “That’s great the way you play and talk with him until he turns his head away to let you know he needs a break.”), and two things to explore when interacting with her baby. Mothers receive three video-feedback sessions conducted at 3-week intervals. Subsequent VID-KIDS sessions follow the same protocol, building on the previous interaction guidance discussions.

### 2.7. VID-KIDS Nurse Interventionist Training and Intervention Integrity

VID-KIDS nurse interventionists completed 25 h of self-paced learning and synchronous (in-person or via Zoom) training sessions facilitated by the primary author. The training comprised seven themed modules, including (1) Introduction to Video-feedback; (2) Parent–Child Relationship Programs Keys to Caregiving Program (Seattle, WA, USA); (3) Infant Engagement and Disengagement Cues; (4) Video-feedback Protocol; (5) Overview of Behaviours of Interest; (6) Video-feedback Case Studies; and (7) Examples of Strengths-Based Feedback. Interventionist training concluded with a reliability check (e.g., a mock video-feedback session) where prospective interventionists had to pass a reliability test, attaining an agreement score of 85%. The lead investigators provided ongoing support and mentoring to nurse interventionists through weekly team meetings to engage in case review, problem-solving, information sharing, and debriefing. Other strategies to maximize implementation fidelity (Carroll et al., 2007) included using a structured intervention fidelity assessment tool to ensure specificity in protocol adherence and ongoing monitoring and evaluating adherence to the video-feedback protocol.

### 2.8. Measures

We collected demographic data pertaining to infant age and gender, maternal age, socioeconomic status (SES), education, and marital status. We also recorded the history of depressive symptoms, details on any treatment for PPD, and whether participants attended any prenatal education or support programmes.

#### 2.8.1. Primary Outcome

Maternal–infant interaction quality was measured via the observational PCITS (Sumner & Spietz, 1994). Considered the best assessment of caregiving quality (Tryphonopoulos et al., 2016), the Teaching Scale is the leading measure of everyday interactions between mothers and infants (up to 36 months). During the Teaching Scale observation, mothers teach their infants a novel, age-appropriate task (e.g., stack blocks). Mother–infant interactions were observed and video-recorded in a 5 min episode without interruption. The Teaching Scale consists of 73 items categorized into six subscales (parental sensitivity to cues, responsiveness to distress, socioemotional growth fostering, cognitive growth fostering, child’s clarity of cues, and child’s responsiveness to their parent). Reliability and validity are well established (Letourneau et al., 2018). For the VID-KIDS pilot (Tryphonopoulos & Letourneau, 2020), internal consistency reliability ranged between 0.77 and 0.84 for the individual subscales and 0.87 for the parent–child total. Coders were blind to group allocation and were required to achieve interrater reliability greater than or equal to 90% on previously scored Teaching Scale materials.

#### 2.8.2. Secondary Outcomes

Maternal depression was measured via the Edinburgh Postnatal Depression Scale (EPDS) (Cox et al., 1987), a 10-item self-report instrument and the standard and most frequently used tool to identify the presence of depressive symptoms in the postpartum period. High scores (>12) are strongly associated with physician diagnosis of Major Depressive Disorder. Maternal anxiety was measured using the State-Trait Anxiety Inventory (STAI) (Spielberger, 1983), a 20-item self-report instrument developed to assess levels of situation-related (state) anxiety. Scores range from 20 to 80, with higher scores indicating higher levels of anxiety. The Parenting Stress Index-Short Form (PSI-SF) was used to assess mothers’ stress in the parenting role (Abidin, 1997). The PSI-SF is a self-report measure comprising 36 items divided into three subscales (Parental Distress, Parent–Child Dysfunctional Interaction, and Difficult Child), yielding a Total Stress score. Parents with a Total Stress score greater than 90 are considered to have clinically significant levels of parenting stress.

Infant developmental outcomes were measured using the Ages and Stages Questionnaire, third edition (ASQ-3) (Squires & Bricker, 2009) and the Ages and Stages Questionnaire: Social–Emotional, second edition (ASQ: SE-2) (Squires et al., 2002) The ASQ-3 is a parent-completed developmental screening instrument that assesses children’s global development across five domains: communication, problem-solving, personal-social, fine motor, and gross motor. There are 21 age-specific questionnaires administered at different age intervals (ranging between 1 and 66 months). Cutoff scores derived from a normative sample are used to determine whether a child’s development in a specific domain appears to be typical (scores above cutoff), needs monitoring (scores near the cutoff), or warrants further assessment (scores below cutoff). The ASQ-3 has demonstrated strong reliability and adequate validity (Squires & Bricker, 2009). For reliability studies, test–retest agreement of classifications (i.e., scores above or below cutoff) for 145 children was 92%; interrater agreement for classifications for 107 children was 93%. Studies examining concurrent validity resulted in sensitivity rates of 86% and specificity of 86% (Chen et al., 2023).

The ASQ: SE-2 (Squires et al., 2002) is a parent-report measure focusing on the social–emotional development of children aged 1 to 72 months. Items assess seven different domains of social–emotional development, including self-regulation, compliance, communication, adaptive functioning, autonomy, affect, and interaction with people. There are nine age-specific questionnaires administered at different age intervals. ASQ: SE-2 scores are summed by adding each item in the specific age interval. The total score is compared with a specific statistically derived cutoff score to determine whether a child’s social–emotional development appears typical, needs monitoring, or warrants further assessment. The ASQ: SE-2 Users’ Guide reported psychometric evidence, including internal consistency reliability (ranging between 0.71 and 0.90) and a test–retest classification agreement (i.e., scores above or below cutoff) for 281 children of 89%.

To assess infant diurnal cortisol patterns, we collected saliva samples at baseline and 2 months post-intervention. The Salimetrics Infant’s Swab (SIS) (Salimetrics SalivaBio L.L.C., 2011; Carlsbad, CA, USA) was used to collect saliva samples from infants, with mothers receiving instructions on how to perform the collection at home. Saliva samples were collected according to the following schedule: upon waking, 30 min after waking, mid-afternoon (around 1400 h), and at bedtime. These times were selected based on the diurnal rhythm of cortisol levels, which typically peak in the early morning and gradually decline throughout the day (Ashman et al., 2002; Pruessner et al., 2003). To encourage adherence to the cortisol collection protocol and record the exact timing of sample collection, MEMS^®^ 6 TrackCap (MEMS^®^, AARDEX; Sion, Switzerland) were provided to participants. Each device consists of a conventional medicine bottle fitted with a special closure that records the time and date of each opening and closing of the container through integrated microcircuitry. This method has been shown to reliably assess participant adherence with a saliva collection protocol and improve compliance since participants know they are being monitored (Hall et al., 2011; Tryphonopoulos & Letourneau, 2020).

### 2.9. Statistical Analysis

Data were checked for outliers and all relevant assumptions. Demographic data were analyzed using means (SD) and percentages as appropriate. Chi-square tests for categorical variables and *t*-tests for continuous variables were used to determine if randomization achieved a balance between the two groups at baseline. Independent sample *t*-tests were used to evaluate differences between the intervention and control groups at post-test and delayed post-test. Paired sample *t*-tests were used to examine changes in outcome measures from baseline to post-test and baseline to the delayed post-test. To test directional hypotheses, statistically significant outcomes were reported using α = 0.05 and one-tailed testing. The Benjamini–Hochberg (BH) (Benjamini & Hochberg, 1995) procedure was applied to control the false discovery rate (FDR), which was set at 5%, for the purpose of assessing the statistical significance of the *t*-tests. Accordingly, for each set of *t*-tests, the highest *p*-value smaller than its corresponding BH critical value (calculated using α = 0.05) was used to determine the corrected critical *p*-value. Effect sizes (Cohen’s *d*) were calculated to quantify the magnitude of the difference between groups. Logarithmic transformation (i.e., base-10 log) was used to normalize data as appropriate for cortisol, which is typically positively skewed (Cruz & Daniel Cruz, 2008). For diurnal cortisol, the area under the curve ground (AUC_G_) and increase (AUCi) calculations were used to facilitate the manageability of multiple time points over the day (Fekedulegn et al., 2007). AUC_G_ reflects the overall concentration of cortisol, and AUCi reflects either an increase or decrease in cortisol concentrations over the course of the day.

## 3. Results

### 3.1. Sample Characteristics

Table 1 presents participant demographic characteristics at baseline. There were no statistically significant differences between group assignment and sociodemographic characteristics (i.e., education, employment status, first language, marital status). All participants lived either in Calgary’s urban centre or the surrounding rural or suburban communities. At randomization, mothers were an average of 32 years old, and infants were 4.98 months old. Most participants spoke English as a first language and were born in Canada. Most participants had a post-secondary degree and were employed. Annual household income ranged between $12,000 and $480,000, with a sample mean of $89,907 (SD = $69,607). Fewer than half (41.33%) of the participants in the intervention group reported attending some form of prenatal education or support programme during their pregnancies, while 58.46% of control participants engaged in such programmes.

### 3.2. Primary Outcome

#### Parent–Child Interaction Quality

Table 2 displays visit #3 (immediate post-test) and visit #4 (delayed post-test) results for the primary outcome of parent–child interaction quality as measured via the PCITS for the combined sample of in-person and Zoom participants. At the immediate post-test, mothers who received the VID-KIDS intervention demonstrated statistically significantly higher scores with respect to sensitivity to cues, cognitive growth fostering, caregiver total scores, and caregiver–child total scores compared to those in the control group. Medium effect sizes for sensitivity to cues (*d* = 0.69), cognitive growth fostering (*d* = 0.74), the caregiver total score (*d* = 0.58), and the caregiver–child total score (*d* = 0.64) were observed, favouring the intervention group.

At the delayed post-test, the intervention group demonstrated statistically significantly higher scores in sensitivity to cues, social–emotional growth fostering, cognitive growth fostering, child’s responsiveness to caregiver, caregiver total scores, child total scores, and caregiver–child total scores compared to those in the control group. Medium positive effects favouring the intervention group were observed for sensitivity to cues (*d* = 0.63), social–emotional growth fostering (*d* = 0.43), cognitive growth fostering (*d* = 0.71), responsiveness to the caregiver (*d* = 0.50), caregiver total score (*d* = 0.66), and the caregiver–child total score (*d* = 0.75).

We conducted additional analyses to compare parent–child interaction quality across the two VID-KIDS intervention delivery methods. Table 3 displays the results for the in-person sample of participants at post-test and delayed post-test, respectively. At post-test, participants who received the VID-KIDS intervention in person demonstrated statistically significantly higher levels of sensitivity to cues, cognitive growth fostering, caregiver total scores, and caregiver–child total scores compared to those in the control group. Medium effect sizes favouring the intervention group were observed for sensitivity to cues (*d* = 0.63), cognitive growth fostering (*d* = 0.65), the caregiver total score (*d* = 0.56), and the caregiver–child total score (*d* = 0.58). At the delayed post-test, the in-person VID-KIDS participants maintained significantly higher levels of sensitivity to cues, cognitive growth fostering, child’s responsiveness to the caregiver, caregiver total scores, and caregiver–child total scores compared to those in the control group. Effect sizes remained consistent with the post-test results.

Table 4 displays the post-test and delayed-post test results for the participants who received the VID-KIDS intervention through Zoom. At post-test, participants who received the VID-KIDS intervention through Zoom demonstrated statistically significantly higher scores in sensitivity to cues, cognitive growth fostering, caregiver total scores, and caregiver–child total scores compared to those in the control group. Large effect sizes were observed for the cognitive growth fostering subscale (*d* = 1.02) and the caregiver–child total score (*d* = 0.80), favouring the intervention group. At delayed post-tests, participants in the intervention group demonstrated statistically significantly higher scores in the following PCITS subscales: sensitivity to cues, social–emotional growth fostering, cognitive growth fostering, responsiveness to caregiver, caregiver total scores and child total scores, and caregiver–child total scores compared to those in the control group. Large effect sizes for social–emotional growth fostering (*d* = 1.01), cognitive growth fostering (*d* = 0.81), the caregiver total score (*d* = 0.85), and the caregiver–child total score (*d =* 0.89) were observed, each favouring the intervention group.

### 3.3. Secondary Outcomes

No statistically significant group differences were observed for any of the secondary outcomes measured in this study (Table 5). These secondary outcomes included maternal depression, maternal anxiety, parenting stress, infant development, and infant cortisol patterns.

## 4. Discussion

### 4.1. Impact of VID-KIDS on Maternal–Infant Interaction Quality

The results of this RCT demonstrate that the VID-KIDS programme effectively improved multiple aspects of mother–infant interaction quality compared to standard care. Mothers who received the VID-KIDS showed significantly higher sensitivity to infant cues, social–emotional growth fostering, cognitive growth fostering behaviours, and overall interaction quality as measured by the PCITS. These positive effects were observed immediately after the intervention and maintained at the 2-month follow-up, suggesting that VID-KIDS had lasting benefits for mothers and infants. The medium to large effect sizes observed for cognitive growth-fostering behaviours and overall caregiver–child interaction quality are particularly noteworthy. These findings suggest that the VID-KIDS programme effectively enhanced mothers’ capacity for providing cognitive stimulation and engaging in positive interactions with their infants. Improvements in these behaviours are manifestly promising, given the critical role of early cognitive stimulation and responsive caregiving in promoting healthy child development (Mateus et al., 2021; Shonkoff & Fisher, 2013). VID-KIDS’ positive impact on maternal sensitivity to infant cues was also encouraging. Maternal sensitivity is a central determinant of secure caregiver–child attachment and children’s later cognitive and socio-emotional development (Madigan et al., 2024). By improving mothers’ abilities to accurately perceive and respond to their infant’s cues, VID-KIDS may also contribute to fostering secure attachment relationships.

Improvements favouring the intervention group were observed across multiple PCITS subscales. Notably, these improvements included the child’s responsiveness to their caregiver. This finding suggests that VID-KIDS had positive impacts not only on maternal behaviours but also on infants’ engagement in the dyadic interaction. The bidirectional nature of these improvements aligns with the transactional model of early relationship development (Sameroff, 2010) and Barnard’s Child Interaction Theory (Barnard & Eyres, 1979). These models share the fundamental premise that child development occurs through continuous, dynamic interactions between the child and their environment, particularly their caregivers. Both the child and the caregiver actively contribute to and shape their interactions over time, creating a reciprocal and mutually influential relationship. As mothers in the intervention group likely adopted more sensitive and responsive caregiving practises, their infants responded with increased engagement. This increased engagement, in turn, likely reinforced and encouraged further positive maternal behaviours, creating a self-reinforcing process. The transactional and Barnard models emphasize the importance of considering both partners when designing and evaluating interventions. By focusing on the mother–infant dyad rather than just on maternal behaviour or infant development in isolation, interventions like VID-KIDS can potentially create more comprehensive and lasting positive changes in early relationships. As the child and caregiver continue to influence each other positively over time, it can lead to cumulative benefits in various aspects of child development, including social, emotional, and cognitive domains (Shonkoff & Fisher, 2013).

This trial demonstrated equivalent effectiveness in improving maternal–infant interaction quality, whether VID-KIDS was delivered in person or via Zoom. This finding suggests that the core components of the VID-KIDS programme—teaching mothers about infant cues and providing supportive interaction guidance through video feedback—can be effectively preserved in a remote format, thus expanding the programme’s potential reach. Remote delivery removes geographical barriers, allowing the VID-KIDS programme to reach families in rural or underserved areas who may not have easy access to in-person services. Remote delivery through videoconferencing platforms such as Zoom also increases convenience for families, potentially improving participation and retention rates (Patterson et al., 2022). The delivery method of the VID-KIDS programme should remain flexible to accommodate the unique circumstances, preferences, and needs of each participating mother to maximize acceptability and impact.

### 4.2. Impact of VID-KIDS on Secondary Outcomes

While the primary focus of the VID-KIDS trial was improving mother–infant interaction quality in the context of PPD, it remains somewhat surprising that group differences were not observed in any of the secondary outcomes. The VID-KIDS programme did not significantly alter maternal depression or anxiety. This finding aligns with previous video-feedback interventions, including our pilot work, which demonstrated improvements in parenting behaviours without corresponding changes in maternal mood (Horowitz et al., 2001, 2013; Tryphonopoulos & Letourneau, 2020). The lack of effect on maternal mental health symptoms may indicate a need for additional intervention components directly targeting mood or for more extended follow-up periods to detect potential downstream effects of improved maternal–infant interactions on maternal wellbeing. It is also worth noting that while the STAI and EPDS are widely used and validated, they may lack the sensitivity to detect subtle changes over a short period, especially in non-clinical samples (Knowles & Olatunji, 2020; Mao et al., 2021). Furthermore, this trial was primarily powered to detect changes in mother–infant interaction quality, potentially limiting our ability to identify smaller yet meaningful changes in secondary outcomes.

Surprisingly, the VID-KIDS programme showed no substantial impact on parenting stress as measured by the PSI-SF (Abidin, 1997). Several factors may explain the lack of response in the current study. The multifaceted nature of parenting stress, which includes aspects beyond parent–child interactions (e.g., parents’ level of distress related to conflicts with a partner, perceptions of social support, and stressors resulting from child rearing) (Lee et al., 2016), may not be fully addressed by an intervention targeting maternal sensitivity and responsiveness. The PSI-SF is a self-report measure, which may be subject to biases. Parents’ perceptions of stress may not immediately align with improvements in their actual interactions with their infants. Additionally, the timing of measurement and the presence of other stressors could mask or limit observable changes in parenting stress levels.

The absence of significant effects on secondary outcomes may also be attributed to the trial’s relatively brief follow-up period. Indeed, while improvements in maternal–infant interaction quality were observed within the study’s timeframe, VID-KIDS’ impacts on infant development and diurnal cortisol levels may require more time to manifest. Furthermore, the 2-month delayed post-test was chosen to balance the need to capture immediate intervention effects with the practical constraints of conducting community-based research. This timeframe aimed to maximize participant retention and minimize the burden on mothers with PPD while still allowing for the assessment of short-term changes in mother–infant interactions.

The lack of significant effects on maternal depression, anxiety, and perceived parenting stress should also be considered within the context of the COVID-19 pandemic, which overlapped with the latter part of the study period (2020–2022). The unique stressors of the pandemic exacerbated mental health challenges for postpartum women, increasing the prevalence of depressive symptoms and heightening stress and anxiety (Kolker et al., 2022; Sahebi et al., 2024). Pandemic-related social isolation reduced access to social support, a known protective factor against these stressors (Kolker et al., 2022). The pandemic ushered in a host of challenges for parents, including social isolation, financial insecurity, and health concerns (Aviles et al., 2024; Kolker et al., 2022), potentially overshadowing the parenting stress-reducing benefits of the intervention. The multifaceted nature of parenting stress during the pandemic, including new challenges like balancing work-from-home with childcare, may have further complicated the assessment of intervention effects. Importantly, while pandemic-related stressors would have affected both intervention and control groups equally, these challenges potentially diminished the ability to detect intervention-specific effects against the backdrop of universally elevated stress levels. Consequently, pandemic-related factors may have contributed to our unexpected secondary outcomes.

The combination of factors discussed above, including measurement limitations, the 2-month follow-up period, and the unprecedented impact of the pandemic, likely contributed to the lack of detectable effects on secondary outcomes. These findings do not diminish the overall rigour of the trial but rather highlight the complex challenges inherent in evaluating interventions in real-world settings. Future research can build upon this foundation, addressing these identified challenges to further refine our understanding of the VID-KIDS programme’s impacts.

### 4.3. Strengths and Limitations

This study was characterized by several strengths. Randomization produced a good balance across the intervention and control groups. The study was adequately powered for the primary outcome, and retention rates were high, making it unlikely that attrition significantly impacted the findings. This observation also implies that participants found the intervention manageable and not overly burdensome. Our primary outcome, maternal–infant interaction quality, was assessed using an observational tool by an expert coder who was blind to group allocation. Additionally, a key strength of this study was our rigorous approach to ensuring VID-KIDS programme fidelity through comprehensive initial training for nurse interventionists, coupled with a structured intervention fidelity tool and ongoing support throughout implementation.

Several limitations of the present study warrant consideration. The face-to-face nature of our intervention precluded blinding of participants, which could introduce performance bias. The sample was relatively homogeneous with respect to high SES and advanced education levels. Given the influence of social determinants of health, particularly socioeconomic disparities in education and income, the effects observed in this trial may not generalize to populations facing greater socioeconomic marginalization (Kim et al., 2024), where the impact of the VID-KIDS programme could be either amplified or diminished. Higher SES participants may have greater access to supportive resources (e.g., healthcare, childcare, social support), potentially influencing intervention outcomes in ways that may not reflect the experiences of more marginalized populations. There may also have been an element of self-selection bias; mothers who agreed to participate in the study may have been more motivated or interested in improving their parenting skills, potentially influencing the outcomes. It should also be noted that most of the secondary outcomes relied on self-report questionnaires, which, although widely used and psychometrically sound, may introduce various forms of reporting bias. However, using established measures such as the STAI and the EPDS remains most appropriate given the current lack of validated observational tools for these constructs, particularly in the context of postpartum mental health. The study included a 2-month post-intervention follow-up; however, a lengthier follow-up (ideally extending several years post-intervention) would be valuable to assess the longer-term impacts of VID-KIDS, particularly on children’s developmental outcomes, providing valuable insights for early childhood intervention strategies.

### 4.4. Implications for Practice and Further Research

The VID-KIDS programme’s success in improving multiple attributes of maternal–infant interaction quality, with medium to large effect sizes, underscores its potential as a valuable tool for promoting healthy early relationships in the context of PPD. Public Health Nurses already play a pivotal role in enhancing the lifelong health of mothers and infants through the care and support they provide families in the early postpartum period. They are uniquely positioned to deliver programmes like VID-KIDS. Many Canadian provinces have well-established public health home-visiting programs, which have the potential to provide existing frameworks that could be leveraged to implement VID-KIDS. For instance, the Healthy Babies, Healthy Children (HBHC) programme is an Ontario-based public health initiative providing home visits by public health nurses and family home visitors to support families. The programme aims to improve child health and development, enhance parent–child relationships, and support parenting capacity. HBHC primarily targets families facing challenges such as mental health issues, limited support systems, or socioeconomic hardships. Building on the foundations of this trial, future research will explore the feasibility of implementing the VID-KIDS programme in “real-world” Public Health settings such as the HBHC programme. This research aims to support the integration and long-term sustainability of VID-KIDS within these existing services, ultimately enhancing support for mothers and infants across Canada.

Recruitment through Public Health Well Child Clinics yielded a relatively diverse sample; however, given that we required English proficiency, the inclusion of newcomers and women whose language of origin was not English was limited. Representation by LBTQ+ caregivers was also limited. Additional research is needed to address these gaps and further validate the programme’s effectiveness. Future studies should explore VID-KIDS’ effectiveness with specific groups, particularly families facing significant social and economic to understand any unique barriers or facilitators to intervention effectiveness in these groups. Moreover, investigating potential moderators of treatment response and examining the longer-term effects of VID-KIDS on maternal mental health and child developmental trajectories will be crucial in refining and optimizing the programme’s impact across diverse populations.

## 5. Conclusions

The VID-KIDS programme is a promising parenting support intervention that enhances mother–infant interaction quality in the context of postpartum depression. Results from this study demonstrate that VID-KIDS was equally effective in both in-person and Zoom formats. The programme’s adaptable delivery options may enhance its accessibility and feasibility for mothers while offering a viable solution for parenting support within resource-constrained settings. Furthermore, VID-KIDS addresses several gaps in current practises of supporting women with PPD, chiefly the need for early intervention during a crucial window for establishing healthy parent–child relationships and the scarcity of practical and scalable positive parenting interventions. Given its demonstrated efficacy, adaptability, and potential to address critical needs in maternal–infant mental health, VID-KIDS represents a notable advancement in evidence-based interventions for PPD, warranting further research, particularly into how this programme can be integrated into standard care practises.

## Figures and Tables

**Table 1:** Baseline Sample Characteristics (*n* = 140).

	Intervention (*n* = 75)			Control (*n* = 65)		
	*n*	Percent or Mean (SD)	Range	*n*	Percent or Mean (SD)	Range
Infant age (months)		4.95 (1.17)	2.72–9.19		5.01 (1.11)	2.61–8.88
Infant sex						
Male	37	49.33%		32	49.23%	
Female	38	50.67.%		35	53.85%	
Infant’s birthweight (kg)		3.26 (0.55)	1.30–4.38		3.13 (0.62)	1.51–4.69
Pre-term birth						
Yes	12	16.0%		9	13.85%	
No	63	84.0%		56	86.15%	
Admitted to neonatal intensive care unit						
Yes	8	10.67%		8	12.31%	
No	67	89.33%		57	87.69%	
Maternal age (years)		32.59 (4.58)	20.58–43.02		31.48 (4.75)	21.64–42.67
Mother’s immigration status						
Born in Canada	56	74.67%		45	69.23%	
Born outside Canada	19	25.33%		20	30.77%	
First language						
English	64	85.33%		55	84.62%	
Not English	11	14.67%		10	15.38%	
Marital status						
Single	5	6.67%		9	13.85%	
Married or common-law	70	93.33%		56	74.67%	
Number of children						
One child	36	48.0%		39	60.0%	
Two children	26	34.67%		20	30.77%	
Three or more children	14	18.67%		6	9.23%	
Education						
Some high school	5	6.67%		6	9.23%	
High school diploma	12	16.0%		15	23.08%	
Post-secondary degree	58	77.33%		44	67.69%	
Employment						
Employed	51	68.0%		51	78.46%	
Unemployed	20	26.67%		13	20.0%	
Student	4	5.33%		1	1.58%	
Duration of depressive symptoms (weeks)		22.06 (20.41)	0–116		17.06 (10.69)	
Attended prenatal education or support programmes						
Yes	31	41.33%		38	58.46%	
No	44	58.67%		27	41.54%	
Prescribed anti-depressant medications						
Yes	20	26.67%		24	36.92%	
No	55	73.33%		41	63.08%	
Currently taking anti-depressant medications						
Yes	13	17.33%		16	24.62%	
No	62	82.67%		49	75.38%	

**Table 2:** Between Group Comparisons of Parent–Child Interaction Quality at Visit #3 (*n* = 131) and Visit #4 (*n* = 125).

		Visit #3			Visit #4			
Parent–Child Interaction Teaching Score	Control Group *n* = 58 Mean (SD)	Treatment Group *n* = 73 Mean (SD)	*p*	*d*	Control Group *n* = 58 Mean (SD)	Treatment Group *n* = 67 Mean (SD)	*p*	*d*
Sensitivity to Cues	8.43 (1.22)	9.16 (0.91)	0.0001 *	0.69	8.95 (0.94)	9.54 (0.91)	0.0001 *	0.63
Response to Child’s Distress	8.78 (2.14)	8.85 (2.10)	0.422	0.04	8.55 (2.10)	8.52 (2.27)	0.470	0.01
Social–Emotional Growth Fostering	7.17 (1.51)	7.38 (1.71)	0.230	0.13	6.74 (1.59)	7.40 (1.51)	0.009 *	0.43
Cognitive Growth Fostering	11.16 (2.74)	12.96 (2.14)	0.0001 *	0.74	11.50 (3.67)	13.46 (1.62)	0.0001 *	0.71
Clarity of Cues	8.10 (1.31)	8.29 (1.09)	0.190	0.16	8.00 (1.08)	8.29 (0.96)	0.059	0.28
Responsiveness to Caregiver	8.53 (2.04)	9.11 (2.14)	0.060	0.28	8.40 (2.32)	9.45 (1.33)	0.003 *	0.50
Caregiver Total Score	35.53 (5.12)	38.36 (4.69)	0.001 *	0.58	35.90 (5.26)	38.93 (3.89)	0.0001 *	0.66
Child Total Score	16.64 (2.65)	17.40 (2.81)	0.059	0.28	16.40 (2.99)	17.71 (2.59)	0.005 *	0.47
Caregiver/Child Total Score	52.17 (6.02)	55.75 (5.22)	0.0001 *	0.64	52.47 (6.19)	56.68 (5.01)	0.0001 *	0.75

* *p* < 0.044 (one-tailed). Note: *p*-values were adjusted using the Benjamini–Hochberg Procedure.

**Table 3:** Between Group Comparisons of Parent–Child Interaction Quality (In-Person Participation) at Visit #3 (*n* = 88) and Visit #4 (*n* = 90).

		Visit #3			Visit #4			
Parent–Child Interaction Teaching Score	Control Group *n* = 37 Mean (SD)	Treatment Group *n* = 51 Mean (SD)	*p*	*d* (Cohen’s *d*)	Control Group *n* = 42 Mean (SD)	Treatment Group *n* = 48 Mean (SD)	*p*	*d*
Sensitivity to Cues	8.49 (1.02)	9.05 (0.92)	0.002 *	0.63	8.83 (1.01)	9.94 (0.94)	0.002 *	0.62
Response to Child’s Distress	8.65 (2.23)	8.67 (2.23)	0.485	0.01	8.26 (2.25)	8.23 (2.39)	0.474	0.01
Social–Emotional Growth Fostering	6.81 (1.33)	7.12 (1.68)	0.180	0.20	6.74 (1.53)	7.06 (1.48)	0.155	0.22
Cognitive Growth Fostering	11.03 (2.56)	12.49 (2.03)	0.002 *	0.65	11.31 (3.75)	13.21 (1.64)	0.002 *	0.67
Clarity of Cues	8.08 (1.14)	8.37 (1.10)	0.114	0.27	8.19 (1.06)	8.29 (1.01)	0.185	0.19
Responsiveness to Caregiver	9.03 (1.92)	9.18 (1.71)	0.351	0.08	8.74 (2.35)	9.56 (1.79)	0.031 *	0.40
Caregiver Total Score	52.08 (5.17)	37.37 (4.21)	0.006 *	0.56	51.98 (5.75)	37.94 (3.83)	0.002 *	0.63
Child Total Score	17.11 (2.55)	17.55 (2.43)	0.206	0.18	16.83 (3.08)	17.85 (2.53)	0.044 *	0.37
Caregiver/Child Total Score	52.08 (5.17)	54.92 (4.72)	0.004 *	0.58	51.98 (5.75)	55.74 (4.93)	0.0001 *	0.72

* *p* < 0.044 (one-tailed). Note: *p*-values were adjusted using the Benjamini–Hochberg Procedure.

**Table 4:** Between Group Comparisons of Parent–Child Interaction Quality (Zoom Participation) at Visit #3 (*n* = 43) and Visit #4 (*n* = 35).

		Visit #3				Visit #4		
Parent–Child Interaction Teaching Score	Control Group *n* = 21 Mean (SD)	Treatment Group *n* = 22 Mean (SD)	*p*	*d*	Control Group *n* = 16 Mean (SD)	Treatment Group *n* = 19 Mean (SD)	*p*	*d*
Sensitivity to Cues	8.83 (1.01)	9.32 (0.89)	0.008 *	0.79	9.25 (0.68)	9.79 (0.79)	0.020 *	0.72
Response to Child’s Distress	8.26 (2.25)	9.00 (2.03)	0.318	0.16	9.31 (1.45)	9.26 (1.79)	0.465	0.03
Social–Emotional Growth Fostering	6.74 (1.53)	7.81 (1.63)	0.352	0.12	6.75 (1.77)	8.26 (1.24)	0.003 *	1.01
Cognitive Growth Fostering	11.31 (3.75)	11.38 (3.09)	0.001 *	1.02	12.00 (3.54)	14.11 (1.41)	0.019 *	0.81
Clarity of Cues	8.19 (1.06)	8.14 (1.59)	0.126	0.04	7.75 (1.13)	8.28 (0.83)	0.063	0.54
Responsiveness to Caregiver	8.74 (2.35)	7.67 (1.98)	0.051	0.51	7.50 (2.03)	9.16 (2.29)	0.016 *	0.76
Caregiver Total Score	51.98 (5.75)	36.52 (6.14)	0.010 *	0.73	37.88 (5.36)	41.42 (2.82)	0.009 *	0.85
Child Total Score	16.83 (3.08)	15.81 (2.68)	0.105	0.40	15.25 (2.46)	17.33 (2.81)	0.014 *	0.79
Caregiver/Child Total Score	51.98 (5.75)	52.33 (7.42)	0.006 *	0.80	53.75 (7.25)	59.06 (4.51)	0.007 *	0.89

* *p* < 0.039 (one-tailed). Note: *p*-values were adjusted using the Benjamini–Hochberg Procedure.

**Table 5:** Between Group Comparisons of Secondary Outcomes at Visit #4.

	*n*	Control Group Mean (SD)	Treatment Group Mean (SD)	*p*	*d*
Parental Outcomes					
EPDS	136	11.65 (5.87)	11.96 (5.08)	0.370	0.06
STAI	131	44.94 (14.02)	46.49 (13.19)	0.258	0.11
PSI-SF	136	81.83 (22.77)	87.27 (19.99)	0.070	0.25
Child Outcomes					
ASQ-3 (Communication Skills)	133	39.17 (16.77)	42.27 (12.50)	0.114	0.21
ASQ-3 (Problem-Solving Skills)	131	46.76 (10.46)	47.40 (10.50)	0.364	0.06
ASQ-3 (Personal-Social Skills)	132	43.00 (10.33)	43.00 (11.52)	0.500	0.00
ASQ-3 (Fine Motor Skills)	131	48.87 (7.45)	48.74 (9.15)	0.466	0.02
ASQ-3 (Gross Motor Skills)	133	42.28 (11.84)	37.58 (15.31)	0.025	0.34
ASQ-SE 2 Total Score	144	0.08 (0.06)	0.08 (0.06)	0.482	0.01
Overall concentration of salivary cortisol	49	6.69 (3.05)	5.95 (2.16)	0.085	0.28
Daily decline of salivary cortisol	49	5.41 (3.52)	4.48 (3.44)	0.060	0.26

Note: EPDS = Edinburgh Postnatal Depression Scale; STAI = State-Trait Anxiety Inventory; PSI-SF = Parenting Stress Index-Short Form; ASQ-3 = Ages and Stages Questionnaires, Third Edition; ASQ-SE 2 = Ages and Stages Questionnaires Social–Emotional. All cortisol values log-transformed. Cortisol measured in µg/L.

## Data Availability

Requests to access the data can be directed to P.T. at ptryphon@uwo.ca.
